# Head-to-Head Comparison of ^68^Ga-Citrate and ^18^F-FDG PET/CT for Detection of Infectious Foci in Patients with* Staphylococcus aureus* Bacteraemia

**DOI:** 10.1155/2017/3179607

**Published:** 2017-10-17

**Authors:** Soile P. Salomäki, Jukka Kemppainen, Ulla Hohenthal, Pauliina Luoto, Olli Eskola, Pirjo Nuutila, Marko Seppänen, Laura Pirilä, Jarmo Oksi, Anne Roivainen

**Affiliations:** ^1^Department of Infectious Diseases, Division of Medicine, Turku University Hospital, Turku, Finland; ^2^Turku PET Centre, University of Turku, Turku, Finland; ^3^Faculty of Medicine, University of Turku, Turku, Finland; ^4^Turku PET Centre, Turku University Hospital, Turku, Finland; ^5^Department of Clinical Physiology and Nuclear Medicine, Turku University Hospital, Turku, Finland; ^6^Department of Endocrinology, Division of Medicine, Turku University Hospital, Turku, Finland; ^7^Department of Rheumatology, Division of Medicine, Turku University Hospital, Turku, Finland

## Abstract

**Purpose:**

This study evaluated the potential of ^68^Ga-citrate positron emission tomography/computed tomography (PET/CT) for the detection of infectious foci in patients with* Staphylococcus aureus* bacteraemia by comparing it with 2-[^18^F]fluoro-2-deoxy-*D*-glucose (^18^F-FDG) PET/CT.

**Methods:**

Four patients admitted to hospital due to* S. aureus* bacteraemia underwent both ^18^F-FDG and ^68^Ga-citrate whole-body PET/CT scans to detect infectious foci.

**Results:**

The time from hospital admission and the initiation of antibiotic treatment to the first PET/CT was 4–10 days. The time interval between ^18^F-FDG and ^68^Ga-citrate PET/CT was 1–4 days. Three patients had vertebral osteomyelitis (spondylodiscitis) and one had osteomyelitis in the toe; these were detected by both ^18^F-FDG (maximum standardised uptake value [SUV_max_] 6.0 ± 1.0) and ^68^Ga-citrate (SUV_max_  6.8 ± 3.5, *P* = 0.61). Three patients had soft tissue infectious foci, with more intense ^18^F-FDG uptake (SUV_max_  6.5 ± 2.5) than ^68^Ga-citrate uptake (SUV_max_  3.9 ± 1.2, *P* = 0.0033).

**Conclusions:**

Our small cohort of patients with* S. aureus* bacteraemia revealed that ^68^Ga-citrate PET/CT is comparable to ^18^F-FDG PET/CT for detection of osteomyelitis, whereas ^18^F-FDG resulted in a higher signal for the detection of soft tissue infectious foci.

## 1. Introduction

Positron emission tomography (PET) with the radiolabelled glucose analogue 2-[^18^F]fluoro-2-deoxy-*D*-glucose (^18^F-FDG) is a sensitive and widely used method to detect inflammation and infection according to the high glucose uptake of activated inflammatory cells. It has an important role in the diagnosis of fever of unknown origin when conventional imaging has failed [1].* Staphylococcus aureus* bacteraemia is a life-threatening condition, and detection and eradication of deep infectious foci are crucial for successful treatment [2]. In previous studies, ^18^F-FDG PET/CT has proven to be a sensitive method for the detection of infectious foci in patients with gram-positive bacteraemia [2, 3].

^68^Ga-citrate has also been shown to be a sensitive and specific tracer for the detection of infectious lesions [4, 5], although only a few human studies using ^68^Ga-citrate PET/CT exist. The biological mechanism of ^68^Ga-citrate accumulation in infectious foci is not fully understood. Once injected the Ga-citrate complex is quickly dissociated into Ga^3+^ and citrate^3−^ within the blood. Then, 99% of the gallium ions are attached to transferrin [6, 7], which accumulates in inflammatory lesions. In addition, it is assumed that some ^68^Ga may attach to bacterial siderophores, lactoferrin inside neutrophils, and free lactoferrin at the site of infection [8]. According to previous studies, ^68^Ga-citrate PET/CT appears to be a sensitive tool for the detection of bone infections [4, 9], although until now it has not been studied in patients with* S. aureus* bacteraemia.

The purpose of this study was to evaluate the potential of ^68^Ga-citrate PET/CT for the detection of infectious foci in patients with* S. aureus* bacteraemia by comparing it with ^18^F-FDG in a head-to-head setting.

## 2. Materials and Methods

### 2.1. Subjects

This study evaluates four consecutive patients who were admitted to hospital due to* S. aureus* bacteraemia. All patients underwent both ^18^F-FDG and ^68^Ga-citrate whole-body PET/CT to detect infectious foci. The study was approved by the institutional ethical review board and all participants signed informed consent. The study was registered as a clinical trial (NCT01878721).

### 2.2. PET/CT

The synthesis of ^68^Ga-citrate was performed with automated synthesis device (Modular Lab, Eckert & Ziegler Eurotope GmbH, Berlin, Germany). ^68^Ga was obtained from a ^68^Ge/^68^Ga generator (IGG-100, 1850 MBq, Eckert & Ziegler Isotope Products, Valencia, CA, USA) by eluting the generator with 6 ml of 0.1 M hydrogen chloride (HCl). ^68^Ga was prepurified through a cationic exchanger (Strata X-C, Phenomenex Inc., Torrance, CA) by eluting with HCl/acetone-solution (800 *μ*l). Acetone was then evaporated by heating at 110°C for 240 s and after cooling of ^68^GaCl_3_, the sterile isotonic sodium citrate solution (4 ml) was added to the reaction vial followed by 240 s reaction time. The product was transferred to the end product vial through a nonpyrogenic 0.22 *μ*m sterile filter and diluted with saline (9 mg/ml, 6 ml). The radiochemical purity of the ^68^Ga-citrate was evaluated by instant thin layer chromatography-silica-gel technique using methanol/acetic acid (9 : 1) as a mobile phase. pH of the product was tested with indicator strips (pH range 2.0–9.0) and sterile filter integrity was assessed by a bubble point test.

Whole-body ^18^F-FDG and ^68^Ga-citrate PET/CT (Discovery VCT, GE Medical Systems) were performed in all patients within 1–4 days. All patients fasted before the ^18^F-FDG scan. The injected radioactivity doses of ^18^F-FDG and ^68^Ga-citrate were 292 ± 68 MBq (range: 227–387 MBq) and 196 ± 37 MBq (range: 158–245 MBq), respectively. PET scanning started at 58 ± 7 min (range: 52–67 min) after ^18^F-FDG injection and 81 ± 23 min (range: 48–100 min) after ^68^Ga-citrate injection. The whole-body PET acquisition (3 min/bed position) was performed following a low dose CT for anatomical reference and attenuation correction. PET images were reconstructed using a 3D maximum-likelihood reconstruction with an ordered-subsets expectation maximization algorithm (VUE Point, GE Healthcare). Visual analysis of the images was performed by an experienced nuclear medicine specialist (J. K.), with the results being reevaluated by the research team for consensus. A positive finding was defined as an abnormal accumulation of ^18^F-FDG or ^68^Ga-citrate indicating infectious foci. ^18^F-FDG and ^68^Ga-citrate uptake in the volumes of interest were quantified and expressed as maximum standardised uptake values (SUV_max_) by normalising the tissue radioactivity concentration for the injected radioactivity dose and the patient's weight. The blood background radioactivity concentration was determined from the left ventricle cavity as SUV_mean_, and the target-to-background ratio (TBR) was calculated as SUV_max,infection_/SUV_mean,blood_.

### 2.3. Statistical Analysis

Results are expressed as mean ± SD and range. A paired *t*-test was used to compare ^18^F-FDG and ^68^Ga-citrate uptake. A *P* value of <0.05 was considered statistically significant.

## 3. Results

^68^Ga-citrate was prepared with high radiochemical purity (≥95%) with pH of 3.0−7.0.

Patient characteristics are presented in Table 1. All patients had a condition predisposing them to infection. In addition, Patient #3 had a cardiac pacemaker. The time interval from hospital admission and initiation of antibiotic treatment to the first PET/CT was 4–10 days, with the second PET/CT scan performed within another 1–4 days. The order of the ^18^F-FDG and ^68^Ga-citrate scans depended on the availability of tracers, with both being performed first in two cases. The mean C-reactive protein (CRP) levels were 89 ± 55 mg/l on the day of ^18^F-FDG PET/CT and 124 ± 118 mg/l on the day of ^68^Ga-citrate PET/CT (*P* = 0.56). The blood background (*n* = 4) SUV_mean_ was 1.4 ± 0.05 for ^18^F-FDG and 2.9 ± 0.88 for ^68^Ga-citrate (*P* = 0.043).

**Figure 1:**
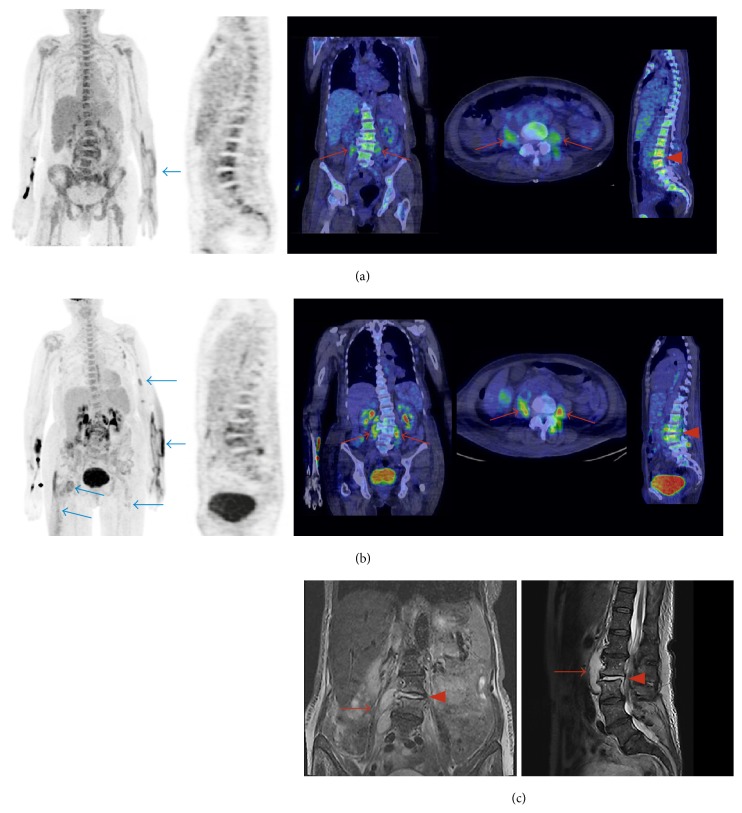
Patient #1 was a 66-year-old woman (weight: 65 kg) who presented at the hospital because of back pain and general symptoms. Both ^68^Ga-citrate (a) and ^18^F-FDG PET/CT (b) showed vertebral osteomyelitis (spondylodiscitis;* red arrowheads*) and abscesses in the iliopsoas and paravertebral area (*red arrows*). These were confirmed by MRI (c). ^18^F-FDG PET/CT also showed other multiple soft tissue infectious foci ((b),* blue arrows*), some of which were not detectable on ^68^Ga-citrate PET/CT ((a),* blue arrow*). The injected radioactivity dose of ^18^F-FDG was 227 MBq and the PET acquisition started 54 min after injection. The injected radioactivity dose of ^68^Ga-citrate was 245 MBq and the PET acquisition started 88 min after injection. MRI sequences were as follows: T2-weighted short inversion time inversion recovery (STIR) on the coronal view image (left) and T2-weighted on the sagittal view image (right).

**Figure 2:**
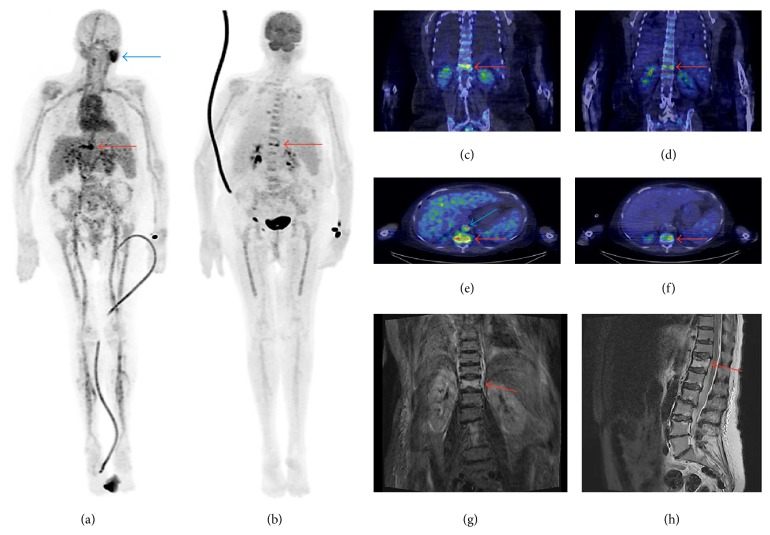
Patient #2 was a 70-year-old woman (weight: 69 kg), with multiple background diseases, who was admitted to hospital because of back pain and high fever. Both ^68^Ga-citrate (a, c, e) and ^18^F-FDG PET/CT (b, d, f) showed vertebral osteomyelitis (spondylodiscitis) in Th12* (red arrows)* and pneumonia in both lungs. MRI showed oedema in Th12 (g, h). ^68^Ga-citrate PET/CT also revealed uptake in the left parotid gland (unspecific; (a),* blue arrow*), neck lymph nodes (reactive), and inferior vena cava (thrombosis; (e),* blue arrow*). There was no ^18^F-FDG uptake in these areas. The injected radioactivity dose of ^18^F-FDG was 279 MBq and the PET acquisition started 50 min after injection. The injected radioactivity dose of ^68^Ga-citrate was 199 MBq and the PET acquisition started 100 min after injection. MRI sequences were as follows: T2-weighted short inversion time inversion recovery (STIR) on the coronal view image (left) and T2-weighted on the sagittal view image (right).

Three patients had vertebral osteomyelitis (spondylodiscitis) and one patient had osteomyelitis of the toe. The osteomyelitic foci were detected by both PET/CT methods (Table 2, Figures 1 and 2) and magnetic resonance imaging (MRI) in the cases of vertebral osteomyelitis and X-ray in the case of osteomyelitis of the toe. In the areas of osteomyelitis (*n* = 4), the SUV_max_ of ^18^F-FDG was 6.0 ± 1.0 (range: 5.3–7.4) and the SUV_max_ of ^68^Ga-citrate was 6.8 ± 3.5 (range: 2.7–11.1, *P* = 0.61). The corresponding TBRs of ^18^F-FDG and ^68^Ga-citrate were 4.4 ± 0.90 and 2.5 ± 1.4, respectively (*P* = 0.015). Three patients had multiple infectious lesions and abscesses in soft tissue (Patients #1, #3, and #4). In these soft tissue infectious foci and abscesses (*n* = 8), the SUV_max_ of ^18^F-FDG (6.5 ± 2.5, range: 3.7–10.6) was significantly higher than that of ^68^Ga-citrate (3.9 ± 1.2, range: 2.1–6.1, *P* = 0.0033; Figure 1). The corresponding TBRs of ^18^F-FDG and ^68^Ga-citrate were 4.6 ± 1.8 and 1.7 ± 0.7, respectively (*P* = 0.00021).

In addition to infectious foci, all of the patients also had other metabolic findings. Three patients (Patients #2, #3, and #4) demonstrated strong uptake of ^68^Ga-citrate in the larger arteries, coincidental to visible atherosclerosis on CT. In the ascending aorta (*n* = 4), the SUV_max_ of ^68^Ga-citrate was 4.7 ± 1.9 (range: 2.0–6.6), whereas the SUV_max_ of ^18^F-FDG was 1.6 ± 0.37 (range: 1.2–2.1, *P* = 0.051). The corresponding TBRs were 1.7 ± 0.72 for ^68^Ga-citrate and 1.2 ± 0.25 for ^18^F-FDG (*P* = 0.17). Patient #1 showed increased splenic uptake of both tracers, which was interpreted as a normal reaction in a septic condition. Patient #2 demonstrated high uptake of ^68^Ga-citrate in an enlarged parotid gland and lymph nodes in the neck (Figure 2(a)), which were not detected on ^18^F-FDG PET/CT. In the absence of clinical symptoms, the enlarged parotid gland was not further studied by MRI or ultrasound, so the observed ^68^Ga-citrate uptake remained unexplained. Patient #2 also demonstrated ^68^Ga-citrate uptake in the inferior vena cava, but not ^18^F-FDG uptake, with thrombosis being confirmed by ultrasonography. Patient #3 had a clear focal accumulation of both ^68^Ga-citrate and ^18^F-FDG in the descending colon, which was subsequently confirmed as a tubular adenoma in a colonoscopy with biopsies. The same patient also demonstrated ^18^F-FDG but not ^68^Ga-citrate accumulation in the caecum without specific findings on colonoscopy or biopsy. Patient #4 had reactive lymph nodes in the neck, which were indicated as being metabolically active by both imaging methods.

## 4. Discussion

We believe that this is the first study to compare ^68^Ga-citrate and ^18^F-FDG PET/CT imaging of infectious foci in patients with* S. aureus* bacteraemia. Our results revealed that ^68^Ga-citrate and ^18^F-FDG are comparable PET tracers for the detection of osteomyelitis. However, the soft tissue infectious foci were clearly better visualized with ^18^F-FDG than ^68^Ga-citrate.

In general, two different tracers must be compared with caution and taking into account their potentially different properties, such as uptake mechanisms and flow/diffusion dependency. In animal models, both ^18^F-FDG and ^68^Ga-chloride have shown increased accumulation in* S. aureus* osteomyelitis, whereas, in healing bones without infection, only ^18^F-FDG accumulation was observed [9]. Our results are in line with the animal studies, in that accumulation of these tracers in patients with osteomyelitis does not differ in the acute phase of* S. aureus* infection. Further studies are warranted to clarify whether ^68^Ga-citrate PET/CT can confirm the healing of osteomyelitis and whether it is superior to ^18^F-FDG for the differentiation of infection from sterile bone inflammation.

We found a difference between ^18^F-FDG and ^68^Ga-citrate PET/CT in the detection of soft tissue infection. Contrary to the clear findings of multiple small infectious foci without abscess formation in ^18^F-FDG PET/CT, such lesions were only slightly visible or not visible at all on ^68^Ga-citrate PET/CT (Patient #1). The difference in SUV_max_ between ^18^F-FDG and ^68^Ga-citrate was also statistically significant. These findings were not controlled by other imaging modalities, but some were also detectable in clinical status (e.g., multiple infectious foci in Patient #1's arm). In this study we used 196 ± 37 MBq for ^68^Ga-citrate PET and started imaging after 81 ± 23 minutes. The observed blood background SUV_mean_ 2.9 + 0.88 may be a strong contributing factor for not visualizing soft tissue infections with ^68^Ga-citrate. It can be hypothesized that lowering of injected radioactivity dose to 100 MBq would reduce blood pool radioactivity and cause less background noise. In order to confirm the effect of a lower ^68^Ga-citrate dose, further studies are warranted. A recent study comparing ^18^F-FDG, ^68^Ga-citrate, and other tracers in a pig-model of hematogenously disseminated* S. aureus* infection reported slightly different results [10]. These findings may be due to the higher blood background radioactivity of ^68^Ga-citrate (Table 2). The slight differences in the production of ^68^Ga-citrate might reflect different results, too.

Owing to its high sensitivity, specificity, and accuracy in the detection of osteomyelitis, MRI is the recommended imaging modality when spondylodiscitis is suspected [11]. MRI provides higher spatial resolution of the spinal cord than PET/CT, which is important if an operation is considered. However, sometimes MRI cannot be obtained because of patient-related reasons (e.g., implanted cardiac devices, claustrophobia). In such cases, ^18^F-FDG PET/CT is the recommended imaging modality [11]. Traditionally, MRI is targeted to the part of the body where the patient has signs or symptoms; however, a previous study [12] showed that around one-third of infectious foci in* S. aureus* bacteraemia are asymptomatic. Thus, these silent infectious foci may pass unnoticed with traditional imaging, while PET/CT scanning provides information on the whole body, and as demonstrated in our cases, multiple infectious foci can be detected simultaneously.

In addition to infectious foci, both ^18^F-FDG and ^68^Ga-citrate PET/CT also revealed other clinically significant findings. In Patient #3, PET/CT findings in the colon led to colonoscopy and the finding of a tubular adenoma. Patient #2 demonstrated an uptake of ^68^Ga-citrate (but not ^18^F-FDG) in the inferior vena cava where thrombosis was later confirmed by ultrasound. This can be related to pooling tracer just proximal to the narrowing caused by the thrombus. More comprehensive studies are needed to explain the differences in the findings of ^68^Ga-citrate and ^18^F-FDG, for example, whether they are ascribed due to infection versus sterile inflammation.

The accumulation of ^68^Ga-citrate in atherosclerotic arteries warrants further studies to determine its importance. Previously, ^68^Ga-chloride uptake has been demonstrated in atherosclerotic lesions in mice [13]. Instead, in the papers presented by Nanni and coworkers [4] as well as Vorster and coworkers [14], the relatively high vascular radioactivity was regarded as a normal biodistribution of ^68^Ga-citrate. The identification of atherosclerosis in 3 patients by ^68^Ga-citrate may be coincidental. One advantage of ^68^Ga-citrate over ^18^F-FDG is that patients are not required to fast before the scan. However, for detection of metastatic endovascular infection the accumulation of ^68^Ga-citrate in atherosclerotic arteries can be regarded as a limitation of ^68^Ga-citrate PET/CT in patients with* S. aureus* bacteraemia.

In the current study, the time intervals between ^18^F-FDG and ^68^Ga-citrate scans were short (1–4 days), and CRP remained at the same levels over these intervals. Neither surgical procedures nor changes to the antimicrobial treatment were made between the two scans. We thus consider that the infectious status of the patients did not differ markedly between ^18^F-FDG and ^68^Ga-citrate studies. However, time interval from commencement of antibiotic treatment to the first PET/CT was 4–10 days and we are not able to exclude the possibility that due to this delay some of the infectious lesions were not detected. We also determined the TBRs, which revealed that the blood radioactivity concentration of ^68^Ga-citrate was higher than that of ^18^F-FDG. Thus, in many foci, the TBRs of ^68^Ga-citrate PET/CT were lower than in ^18^F-FDG PET/CT. In general, the number of patients is small, which can be regarded as a limitation of this study.

## 5. Conclusion

^68^Ga-citrate and ^18^F-FDG are comparable PET tracers for the imaging of osteomyelitis in patients with* S. aureus* bacteraemia. Further studies are warranted to clarify whether ^68^Ga-citrate PET/CT can detect osteomyelitis caused by other pathogens and whether it can assess the healing of infectious osteomyelitis. For the detection of soft tissue infectious foci, ^18^F-FDG PET/CT shows higher intensity than ^68^Ga-citrate PET/CT but the effect of lower ^68^Ga-citrate dose should be verified by further studies.

## Figures and Tables

**Table 1:** Characteristics of the study patients.

Patient number	Age (years)	Gender	Comorbidities	Time from starting antibiotics to ^18^F-FDG PET/CT (days)	Time from starting antibiotics to ^68^Ga-citrate PET/CT (days)	CRP on ^18^F-FDG PET/CT (mg/l)	CRP on ^68^Ga-citrate PET/CT (mg/l)	Complications from *S. aureus* bacteraemia based on routine exams, PET/CTs, and follow-up
(1)	66	F	Atopic eczema	10	11	92	109	Meningitis, vertebral osteomyelitis, paravertebral abscesses, multiple infectious foci in soft tissues
(2)	70	F	Liver cirrhosis, type 2 diabetes, generalised atherosclerosis, HTN	6	5	14	14	Vertebral osteomyelitis, pneumonia
(3)	66	M	Cardiomyopathy, HTN, asthma, cardiac pacemaker	5	4	105	291	Osteomyelitis in toe and feet
(4)	86	M	COPD, immunosuppressive medication due to chronic dermatology disease	8	12	146	83	Vertebral osteomyelitis, septic arthritis, multiple infectious foci in soft tissues

F, female; M, male; HTN, hypertension; COPD, chronic obstructive pulmonary disease; CRP, C-reactive protein.

**Table 2:** Quantitative ^18^F-FDG and ^68^Ga-citrate PET/CT findings.

Patient number	Visually active findings	^18^F-FDG		^68^Ga-citrate	
		SUV_max_	TBR	SUV_max_	TBR
	*Infectious foci*				
(1)	Blood background^a^	1.4	—	2.1	—
	Vertebral osteomyelitis	6.1	4.4	7.3	3.5
	Psoas/paravertebral abscess	10.6	7.6	6.1	2.9
	Left elbow abscess	9.4	6.7	4.0	1.9
	Three soft tissue infectious foci in left arm	7.3; 6.3; 5.4	5.2; 4.5; 3.9	5.0; 3.6; 3.5	2.4; 1.7; 1.7
	Soft tissue infectious focus in left gluteus	3.7	2.6	2.1	0.8
(2)	Blood background^a^	1.3	—	2.9	—
	Vertebral osteomyelitis	7.4	5.7	11.1	3.8
	Pneumonia, right lung	2.7	2.1	3.7	1.3
	Pneumonia, left lung	3.9	3.0	3.1	1.1
(3)	Blood background^a^	1.4	—	2.4	—
	Septic arthritis and osteomyelitis, I toe	5.3	3.8	2.7	1.1
	Soft tissue infectious focus, I toe	4.6	3.3	2.9	1.2
(4)	Blood background^a^	1.4	—	4.1	—
	Vertebral osteomyelitis	5.3	3.8	5.9	1.4
	Shoulder abscess	4.5	3.2	4.2	1.0
	Septic arthritis, glenohumeral joint	5.0	3.6	2.5	0.6
	Septic arthritis, sternoclavicular joint	7.7	5.5	6.2	1.5

	*Other metabolic findings*				
(1)	Active spleen	2.9	2.1	2.7	1.3
	Ascending aorta	1.6	1.1	2.0	1.0
(2)	Parotid, unexplained	1.8	1.4	9.4	3.2
	Reactive lymph nodes, neck right side	2.5	1.9	5.6	1.9
	Inferior vena cava, thrombosis	2.5	1.9	8.6	3.0
	Ascending aorta	1.6	1.2	6.6	2.3
(3)	Caecum, unspecific uptake	12.6	9.0	1.7	0.7
	Descending colon, tubular adenoma	9.7	6.9	7.8	3.3
	Ascending aorta	2.1	1.5	5.4	2.3
(4)	Reactive lymph nodes, neck right side	6.8	4.9	4.6	1.1
	Reactive lymph nodes, neck left side	6.6	4.7	3.7	0.9
	Ascending aorta	1.2	0.9	4.6	1.1

^a^Determined from heart left ventricle cavity, SUV_mean_; TBR, target-to-background ratio (SUV_max,infection_/SUV_mean,blood_).
